# The efficacy and safety of warming acupuncture and moxibustion on rheumatoid arthritis: A protocol for a systematic review and meta-analysis

**DOI:** 10.1097/MD.0000000000021857

**Published:** 2020-08-21

**Authors:** Jingwen Shang, Wei Fan, Zhongqing Dou, Linlin Wu, Bibo Lu, Junhui Qian

**Affiliations:** a Chengdu Eighth People's Hospital; b Tianjin University of Traditional Chinese Medicine, Tianjin; c Hospital of Chengdu University of Traditional Chinese Medicine, Chengdu, Sichuan Province, China.

**Keywords:** meta-analysis, protocol, rheumatoid arthritis, systematic review, warming acupuncture and moxibustion

## Abstract

**Background:**

Rheumatoid arthritis (RA), as an autoimmune disease, can eventually lead to joint deformity and loss of function, seriously reduce the quality of life of patients and increase economic burden. As a traditional Chinese therapy, warming acupuncture and moxibustion is safe, economical, and has few side effects. At present, some studies have shown that warming acupuncture and moxibustion has a certain effect on RA, but there is no evidence of evidence-based medicine. The purpose of this study was to evaluate the efficacy and safety of warming acupuncture and moxibustion in the treatment of rheumatoid arthritis.

**Method:**

Randomized controlled trials of warming acupuncture and moxibustion treating RA will be searched in the databases including PubMed, EMBASE, the Cochrane library, Web of science, China National Knowledge Infrastructure (CNKI), WanFang, the Chongqing VIP Chinese Science and Technology Periodical Database (VIP), and China biomedical literature database (CBM) from inception to July, 2020. In addition, Baidu, Google Scholar, International Clinical Trials Registry Platform, and Chinese Clinical Trials Registry will be searched to obtain the gray literature and relevant data that have not yet been published. Two qualified researchers will extract data and assess the risk of bias from included studies independently. Statistical analysis is performed in RevMan 5.3 software.

**Results:**

The primary outcome is symptom evaluation including morning stiffness, pain, and joint swelling. The number of joints affected by RA, Rheumatoid factor (RF), erythrocyte sedimentation rate (ESR), C reactive protein (CRP), anti-cyclic peptide containing citrulline (A-CCP), and adverse effects, will be evaluated as secondary outcomes.

**Conclusions:**

This study will compare the efficacy and safety of warming acupuncture and moxibustion with common acupuncture in the treatment of RA, providing reliable evidence for clinical application.

**OSF Registration number:**

DOI 10.17605/OSF.IO/C8RY9.

## 1 Introduction

Rheumatoid arthritis (RA) is a common form of inflammatory autoimmune disease with unknown etiology. Bone erosion and destruction, cartilage degradation, synovial inflammation are 3 major pathways of RA pathology.^[1]^ This disease eventually leads to joint malformations and loss of function. Meanwhile, RA patients are often complicated with cardiovascular diseases, brittle fractures, malignant tumors, depression, etc, which not only affects the prognosis of RA patients, but also raises the mortality. The epidemiological survey shows that the current incidence of RA is 0.5% to 1% in the world and 0.42% in mainland of China. The total population of RA is about 5 million, among which women are more susceptible to RA than men and the ratio of males to females is about 1:4.^[2]^ With the disease progressing, the disability rate gradually increases. RA not only reduces patients’ physical function, quality of life, and social participation, but also adds huge economic burden to families and society.

The treatment methods of RA in western medicine involve drugs, gene therapy and surgical treatment. Drugs include nonsteroidal anti-inflammatory drug (NSAIDs), disease modifying anti-rheumatic drugs (DMARDs), and corticosteroids. Currently, the preferred drug at home and abroad is conventional synthetic DMARDs, for example, Methotrexate, is the typical first-line conventional therapeutic agent in RA. Furthermore, conventional synthetic DMARDs combined with biological or targeted synthesis of DMARDs is used to treat RA while the conventional DMARDs combined with corticosteroids is used to treat moderately or highly active RA. However, drugs have some limitations like impassive to RA, severe side effects, chronic treatment, frequent medication, lack of permanent curative response, the abuse of corticosteroids and relapse after withdrawal of drug. But traditional acupuncture and moxibustion therapy for RA can provide an alternative to avoid above problems.^[1,2]^

As a popular complementary and alternative therapy, acupuncture has a good clinical effect all over the world. Traditional acupuncture therapy includes needling and moxibustion. Warm acupuncture and moxibustion is a therapeutic combination of both. After obtaining Qi through acupuncture, the needle should be kept at an appropriate depth, and the moxa should be fixed on the needle handle and ignited, so that the heat can be transmitted to the acupuncture point through the needle body. On the basis of acupuncture, the heat can warm the meridians and promote Qi and blood circulation. Studies^[3]^ suggest that acupuncture or moxibustion interventions may have a positive effect in pain relief, physical function in RA patients. Acupuncture exerts anti-inflammatory and analgesic effects via elevating anti-inflammatory cytokine level and reducing pro-inflammatory cytokine level and regulating Th1/Th2 balance, which reflects the dual regulatory effect of acupuncture.^[4]^ The moxibustion can regulate the body's immune mechanism and inflammatory factor content, relieve pain. However, there is no literature to systematically evaluate the efficacy and safety of warm acupuncture on RA.^[5]^ Therefore, the purpose of this systematic review and meta-analysis is to evaluate the efficacy and safety of warm acupuncture and moxibustion in RA patients and provide reliable evidence for clinical application.

## 2 Methods

### 2.1 Study registration

This protocol of systematic review and meta-analysis has been drafted under the guidance of the preferred reporting items for systematic reviews and meta-analyses protocols (PRISMA-P). Moreover, it has been registered on open science framework (OSF) on July 17, 2020. (Registration number: DOI 10.17605/OSF.IO/C8RY9).

### 2.2 Ethics

Ethical approval is not required because there is no patient recruitment and personal information collection, and the data included in our study are derived from published literature.

### 2.3 Inclusion criteria for study selection

#### 2.3.1 Types of studies

We will collect all available randomized controlled trails (RCTs) on warming acupuncture treatment for RA, regardless of blinding, publication status, region, but Language will be restricted to Chinese and English.

#### 2.3.2 Type of participants

All participants diagnosed as RA will be included irrespective of their country, age, race, and sex.

#### 2.3.3 Type of interventions

In the experimental group, RA patients will be treated with warm acupuncture. In the control group, RA patients will receive common acupuncture. The above 2 groups of manipulation, acupoints and the treatment courses are not restricted.

#### 2.3.4 Type of outcome measures

(1) Primary outcome: symptom evaluation including morning stiffness, pain and joint swelling. The number of swelling joints affected by RA; the number of painful joints affected by RA; the duration of morning stiffness.
(2) Secondary outcomes: pain visual analog scale (VAS) score; rheumatoid factor; erythrocyte sedimentation rate; C reactive protein; anti-cyclic peptide containing citrulline; adverse effects.

### 2.4 Exclusion criteria

(1) If the study is published repeatedly, the one with the most complete data will be included;
(2) Studies with missing data, and can’t get the data after contacting the author;
(3) Studies with obviously wrong data;
(4) If an incorrect random method is used, the literature will be excluded.

### 2.5 Search strategy

Randomized controlled trials of warming acupuncture and moxibustion for the treatment of RA will be searched in the databases including PubMed, EMBASE, the Cochrane library, Web of science, China National Knowledge Infrastructure (CNKI), WanFang, the Chongqing VIP Chinese Science and Technology Periodical Database (VIP), and China biomedical literature database (CBM) from inception to July, 2020. In addition, Baidu, Google Scholar, International Clinical Trials Registry Platform, and Chinese Clinical Trials Registry will be searched to obtain the gray literature and relevant data that have not yet been published. The search terms are as follows: “acupuncture,” “moxibustion,” “warming acupuncture,” and “rheumatoid arthritis,”, etc. The search strategy (PubMed) is shown in Table 1.

**Table 1:**
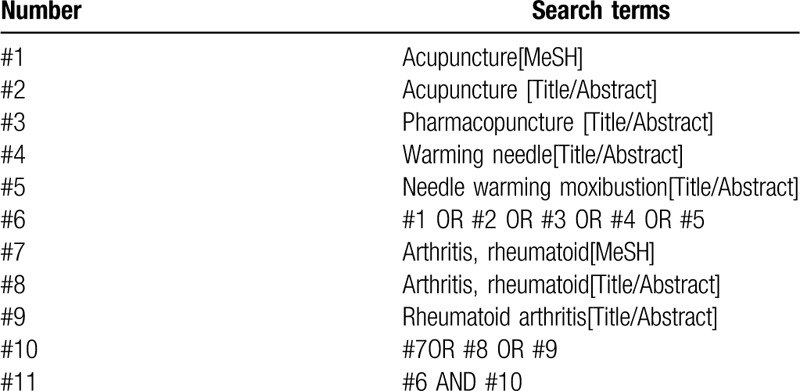
Search strategy in PubMed database.

### 2.6 Data extraction

**Figure 1:**
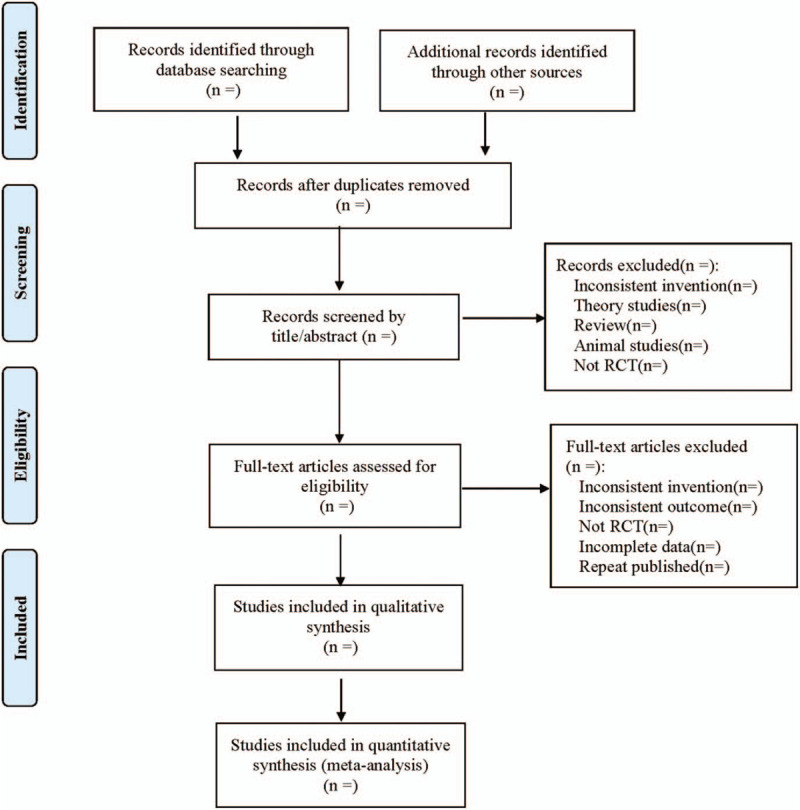
Flow diagram.

Searches will be conducted in the above databases according to guidance from The Cochrane Collaborative Network System Evaluator's Manual Version 5.0. Two researchers will select the literature by reading the title and abstract separately. Related studies will be initially included and imported into Endnote X7 (developed by The National Institute of Scientific Information) for removal of duplications. Then, by reading the full text of the article, new screening is carried out by referring to the inclusion criteria and exclusion criteria. Two qualified researchers will extract data from included studies independently. Any inconsistent views will be solved by a third researcher through discussion. The following information will be extracted: basic information of included studies: study title, first author, publication year, and so on; basic characteristics: sample size, age, sex ratio, and the course of disease between experimental group and control group; Interventions: intervention measure, intervention time, frequency, treatment course; the entry of risk of bias assessment; the outcomes and relevant measurement data. The literature screening process is shown in Fig. 1.

### 2.7 Quality assessment

The risk of bias for each eligible study will be assessed by 2 researchers respectively according to the Cochrane Collaboration's tool including 7 terms. According to these criteria (random sequence generation, allocation concealment, blinding, incomplete data, selective result reports, and other bias), risk of bias is classified into the following levels: unclear, low, and high risk of bias. Any divergences will be solved through discussion by a third researcher.

### 2.8 Statistical analysis

#### 2.8.1 Data synthesis

Statistical analysis is performed in RevMan 5.3 software (developed by the UK's International Cochrane Collaboration). For dichotomous outcome data, the risk ratio (RR) with 95% confidence intervals (CIs) is calculated. For continuous outcome data, if the measurement tool or measurement index unit is the same, the weighted mean difference (WMD) with 95% CIs is adopted; if the measurement tool or measurement index unit is different, the standard mean difference (SMD) with 95% CIs is used. Statistical heterogeneity will be checked by *P* value and *I*^2^ statistics. If *P* ≥ .1, *I*^2^ ≤ 50%, there is no significant heterogeneity; thus the fixed-effect model is used. When *P* < .1, *I*^2^ї¿≥ 50%, the sources of heterogeneity are analyzed, and subgroup analysis may be performed. If there is statistical heterogeneity between studies but no clinical heterogeneity, the random-effect model is selected to analyze. Otherwise, we exclude the study from meta-analysis.

#### 2.8.2 Dealing with missing data

When occurring missing, incomplete, and unclear data, we will contact the corresponding author to obtain it. If data are not acquired, we will exclude this literature.

#### 2.8.3 Subgroup analysis

Subgroup analysis will be constructed based on the age of patients, the course of disease, and the intervention time.

#### 2.8.4 Sensitivity analysis

In order to verify the stability of the results of each outcome indicator, we adopt the one-by-one elimination method for sensitivity.

#### 2.8.5 Assessment of reporting biases

When >10 studies are included, funnel plots are used to detect reporting bias. Egger and Begg tests are used to quantitatively assess potential publication bias.

## 3 Discussion

In Chinese medicine, rheumatoid arthritis belongs to the category of “Bi Zheng” (Arthralgia syndrome). The basic pathogenesis of the disease is the sense of deficiency and stagnation of meridians, and the key of its pathological changes is that phlegm, dampness, and blood stasis block the meridians. With the prolongation of the course of disease, the rate of disability is rising, which seriously affects the quality of life of patients and aggravates the economic burden. Currently, traditional Chinese medicine has a variety of treatment methods for this disease, especially acupuncture and moxibustion. Compared with oral drugs, acupuncture and moxibustion have fast onset and no side effects, and it becomes a complementary and alternative therapies for RA.^[6]^ As a remedy of combining acupuncture and moxibustion, its functions of warming meridians to expel cold, activating blood circulation and qi, eliminating stasis and dispersing knot are in accordance with the pathogenesis of Arthralgia syndrome. Research has proved warm needle moxibustion can relieve inflammatory reactions of RA, which may be associated to its effect in modulating NF-κB signaling in the synovial tissue.^[6–8]^ In addition, clinical trials have proved that warm acupuncture and moxibustion in the treatment of RA is more effective than conventional acupuncture,^[9,10]^ but there is no systematic evaluation or meta-analysis on the efficacy and safety of warm acupuncture and moxibustion in the treatment of RA, so this study is of great significance. However, the study has some limitations. There are certain heterogeneity due to the difference of acupoints. And only studies published in English and Chinese are retrieved, so important studies published in other languages may be missed.

## Author contributions

**Data collection:** Jingwen Shang and Wei Fan.

**Funding support:** Haifa Qiao.

**Literature retrieval:** Wei Fan and Zhongqing Dou.

**Software operating:** Linlin Wu and Bibo Lu.

**Supervision:** Bibo Lu and Haifa Qiao.

**Writing – original draft:** Jingwen Shang and Wei Fan.

**Writing – review & editing:** Jingwen Shang and Haifa Qiao.
